# Supplementary material for: Interpretable machine learning for chronic kidney disease prediction: Insights from SHAP and LIME analyses
Source: PLoS One. 2026 Feb 26;21(2):e0343205. doi: 10.1371/journal.pone.0343205 (PMC12944776; doi:10.1371/journal.pone.0343205)
Supplement: Attachment — Submitted filename: reviewer.pdf [file pone.0343205.s001.pdf]

Response to Reviewers and Academic Editor

Manuscript Title: Interpretable Machine Learning for Chronic Kidney Disease Prediction: Insights from SHAP and LIME Analyses
Manuscript ID: PONE-D-24-28794

Dear Editorial Team,

We would like to express our sincere appreciation for the thorough and constructive feedback provided on our manuscript. We have carefully considered each comment and have made corresponding revisions to enhance the quality and clarity of our work. Below, we provide a detailed, point-by-point response to each of the reviewers' comments.

Reviewer #1

1. **Introduction:**
    - **Comment:** Defining CKD at the first mention would improve clarity.
    - **Response:** We appreciate this insightful suggestion. To improve clarity, we have revised the Introduction section to include a clear definition of Chronic Kidney Disease (CKD) at its first mention. This adjustment ensures that all readers, regardless of their familiarity with the subject, can fully understand the content from the outset.
2. **Methodology:**
    - **Comment:** The explanation of SHAP and LIME for model interpretability is well-done. However, including a pseudo-code or flowchart for the proposed methodology would enhance understanding.
    - **Response:** Thank you for your positive feedback and valuable suggestion. We have incorporated the following enhancements:
        - **Flowchart:** A detailed flowchart (Figure 2) has been added to visually represent the entire methodology, from data collection to the application of explainable AI techniques.
        - **Pseudo-code:** We have included a pseudo-code representation of the key steps to improve reproducibility and provide clearer insights into our approach.

      These improvements are aimed at enhancing the transparency and comprehensibility of our methodology.
    - **Comment:** Authors should provide more details on the extent of missing data and the specific imputation strategy used.

- **Response:** Thank you for highlighting this important aspect. We have expanded the "Data Preprocessing" section to include a detailed account of the extent of missing data in Dataset 1. Specifically, *TriglyceridesBaseline* had 6 missing values, and *HgbA1C* had 15 missing values. We utilized Multiple Imputation by Chained Equations (MICE) as the imputation strategy, which preserves the relationships within the data through an iterative process. These revisions have been made to ensure a comprehensive understanding of our data handling procedures.

- **Comment:** The application of SMOTE for class balancing is well-justified, but the authors should discuss potential drawbacks of this technique, such as overfitting, and how they addressed these concerns.

- **Response:** We appreciate your detailed feedback on the use of SMOTE. To address potential concerns, we have included an explanation of the SMOTE technique, supported by a mathematical formula, to clarify its application for class balancing. Additionally, we have discussed the potential for overfitting and our mitigation strategies, which include the implementation of stratified k-fold cross-validation and hyperparameter tuning. These updates have been added to the "Class Balancing" section.

- **Comment:** The choice of machine learning algorithms (logistic regression, random forest, decision trees, naive Bayes, XGBoost, LightGBM) is suitable for comparison. The use of hyperparameter tuning via grid search is also appropriate. However, it would be beneficial to provide more details on the computational resources and time required for training these models.

- **Response:** Thank you for your insightful feedback. We agree that understanding the computational demands of our approach is crucial. In response, we have added a new subsection in the "Results" section detailing the computational environment used for training the models, as well as the training times for each model. This addition provides a clearer picture of the practical considerations involved in deploying these models.

- **Comment:** Statistical Analysis - The authors used appropriate performance metrics (accuracy, ROC-AUC, precision, recall, F1-score). The use of stratified k-fold cross-validation (10-fold) is robust and helps prevent overfitting. However, details on the variance or confidence intervals of these metrics across folds would provide a better understanding of model stability.

- **Response:** We are grateful for your suggestion to enhance the robustness of our statistical analysis. In the revised Statistical Analysis section, we have included variance and 95% confidence intervals for performance metrics (accuracy, ROC-AUC, precision, recall, F1-score) across the 10-fold cross-validation. This addition offers a more comprehensive understanding of the stability and reliability of our models.

3. **Figures & Tables:**

- **Comment:** Figures 3, 4, 5, 6, and 7, which include ROC curves, SHAP summary plots, and LIME explanations, are well-presented. However, some figures are densely packed with information. Consider breaking down these figures or providing zoomed-in views of key sections to enhance readability.

- **Response:** Thank you for your constructive feedback on our figures. We have revised Figures 3, 4, 5, 6, and 7 by breaking them down and providing zoomed-in views. These adjustments have been made to enhance readability and ensure that key information is presented more clearly.
- **Comment:** Tables summarizing performance metrics are clear, but including standard deviations or confidence intervals for these metrics would improve the presentation of the results.
- **Response:** We appreciate your suggestion to enhance the clarity of our tables. We have updated the tables summarizing performance metrics to include standard deviations and 95% confidence intervals. This addition provides a more nuanced understanding of the variability and reliability of our results.

4. **Results:**

- **Comment:** The results section clearly shows that XGBoost outperforms other models. However, the performance differences are not always substantial. It would be useful to discuss why XGBoost, despite being more complex, offers only marginal improvements over simpler models like logistic regression in certain cases.
- **Response:** Thank you for this astute observation. We have added a discussion in the Results section to explain that the marginal improvements offered by XGBoost may be attributable to the linear nature of the data, where simpler models like logistic regression perform effectively. XGBoost's complexity is better leveraged in datasets with non-linear relationships, which may not be prominent in this case.
- **Comment:** The authors should also address potential biases introduced by the datasets, such as class imbalance even after SMOTE application and any residual confounding variables.
- **Response:** We appreciate your attention to potential biases. We have included a discussion acknowledging that SMOTE may introduce synthetic data that does not fully capture real-world complexity, and we have noted the possibility of residual confounding variables that could affect model predictions. These considerations have been incorporated into the revised manuscript to provide a more comprehensive evaluation of our results.

5. **Limitations and Future Work:**

- **Comment:** Authors should elaborate on the impact of potential data quality issues, such as measurement errors or inconsistent data collection practices.
- **Response:** We agree with the importance of addressing data quality issues. We have expanded the Limitations section to discuss the impact of measurement errors and inconsistent data collection practices on model performance. Additionally, we have suggested that future research focus on improving data quality through rigorous collection protocols and advanced preprocessing techniques.

6. **Basic Changes to be Addressed:**

- **Comment:** Check abbreviation (defined at first use), typographical, and reference formatting.
- **Response:** We have carefully reviewed and corrected all abbreviations, typographical errors, and reference formatting in accordance with the journal's guidelines. We appreciate your attention to these details, which help improve the overall presentation of our manuscript.

Reviewer #2

1. **Logic of the Paper and Novelty:**
    - **Comment:** The rationale for choosing the six machine learning methods in the paper should be clearly explained, and their advantages outlined.
    - **Response:** We appreciate your feedback on the need for clarity in our methodological choices. We have revised the Introduction to provide a clear rationale for selecting the six machine learning models used in our study, highlighting each model's strengths and relevance to clinical applications. We have also identified specific research gaps, emphasizing the need for a comprehensive comparison of these models in CKD prediction. The novelty of our study is articulated by integrating SHAP and LIME for model interpretability, critically analyzing relevant works, and demonstrating the clinical implications of our findings. These revisions strengthen the logical flow and underscore the significance of our research.
2. **Formatting Issues:**
    - **Comment:** Number the titles at each level, right-align the formula numbers, centrally align table contents, differentiate chart legends, and further standardize the format of the references.
    - **Response:** Thank you for your detailed feedback. We have implemented all suggested formatting changes as follows:
        - Numbered all titles and subtitles for clarity.
        - Right-aligned all formula numbers for consistency.
        - Centrally aligned table contents for improved readability.
        - Enhanced chart legends for better differentiation and understanding.
        - Standardized the reference format in accordance with the journal's guidelines.

      We believe these changes significantly improve the presentation and readability of our manuscript.
3. **Explanation of Symbols in Formulas:**
    - **Comment:** The corresponding explanation of the symbols in some formulas should be supplemented.
    - **Response:** Thank you for highlighting this aspect. We have supplemented the explanations for the symbols used in the formulas to ensure clarity and comprehension. This enhancement is intended to make our manuscript more accessible to readers who may not be familiar with these symbols.

4. **Data Normalization and Partitioning:**
   - **Comment:** The modality for partitioning the data set into training and testing data should be explained, and data normalization should be performed before model adoption to improve accuracy.
   - **Response:** We appreciate your valuable suggestions. The methodology section has been restructured to include detailed explanations for each step, including data preprocessing, splitting, and model evaluation. Specifically, we have added a step for data normalization before training the models, as recommended. This ensures that the training and testing datasets are on the same scale, thereby improving model accuracy and performance. These changes align our methodology with best practices in machine learning.

5. **Major Findings:**
   - **Comment:** The major findings of the study should be provided in bullet points.
   - **Response:** Thank you for this suggestion to improve clarity. We have revised the manuscript to present the major findings in bullet points, ensuring that the key contributions of our study are clearly highlighted and easily accessible to readers.

6. **Future Research Considerations:**
   - **Comment:** Future research considerations should be suggested based on the findings of the research.
   - **Response:** We appreciate your forward-looking suggestion. We have included a section on future research considerations in the revised manuscript, highlighting potential areas for further investigation based on our findings. This addition provides a clear direction for subsequent studies and aims to extend the impact of our research.

Reviewer #3

1. **Model Validation and Comparison:**
   - **Comment:** The paper lacks sufficient validation, as it does not utilize an adequate number of models to justify the results through comprehensive comparison. A broader model comparison, including neural networks, is necessary to strengthen the findings.
   - **Response:** Thank you for your insightful feedback. Recognizing the importance of comprehensive model validation, we have expanded our analysis to include additional neural network models. This broader comparison strengthens our findings by demonstrating how our models perform relative to a wider range of algorithms, thus providing a more robust evaluation of our approach.

2. **Execution Time:**

- **Comment:** The authors have not mentioned the time taken by each model during execution, which is crucial for evaluating the efficiency and practicality of the proposed approach.
- **Response:** We appreciate your observation regarding the importance of execution time in evaluating model efficiency. To address this, we have included a new subsection in the "Results" section detailing the execution time for each model, alongside the computational environment used. This information provides a comprehensive assessment of both the accuracy and efficiency of our models, which are critical considerations in practical applications.

3. **Explanation of SMOTE and MICE:**

- **Comment:** The paper doesn't explain what SMOTE (Synthetic Minority Over-sampling Technique) is or how it helped balance the data. A brief explanation would be helpful. The same goes for MICE (Multiple Imputation by Chained Equations); a short description of how it was used and its impact on the data would make the methodology clearer.
- **Response:** Thank you for pointing out the need for additional clarity. We have included brief explanations of both SMOTE and MICE in the revised manuscript. SMOTE was used to address class imbalance by generating synthetic examples for the minority class, thereby preventing bias towards the majority class. For MICE, we provided a description of its role in handling missing data through an iterative process that estimates missing values based on variable relationships. These clarifications have been added to the "Data Preprocessing" section to provide a more complete understanding of our methodology.

Reviewer #4

1. **Dataset Characteristics in Abstract:**

- **Comment:** The use of XGBoost and comparison with traditional algorithms is commendable, the abstract should provide more insight into the dataset characteristics, such as size, demographic diversity, and any potential biases. This information is crucial for evaluating the generalizability and robustness of the findings.
- **Response:** Thank you for your positive feedback and valuable suggestion. We have revised the abstract to include more detailed information about the datasets used in the study. Specifically, we have added details on the size of the datasets, the demographic diversity of the populations represented, and a brief discussion of potential biases that could affect the generalizability and robustness of our findings. These additions aim to provide a clearer understanding of the context in which our models were developed and evaluated.

2. **Application of SHAP and LIME:**

- **Comment:** The integration of SHAP and LIME for interpretability mentioned in the manuscript is a strong point. However, it would be helpful to

include a brief description of how these methods were applied and any limitations encountered in their application. This could clarify the extent to which the interpretability achieved can be trusted and utilized in clinical practice.

- **Response:** Thank you for recognizing the strengths of our interpretability methods. In response to your suggestion, we have added a brief description detailing how SHAP and LIME were applied to our XGBoost model. We have also discussed the limitations encountered in their application, specifically addressing the contexts in which the interpretability achieved can be trusted and effectively utilized in clinical practice. These additions aim to provide greater clarity and ensure that the interpretability results are both reliable and actionable.

3. **Accuracy and ROC-AUC Scores:**

   - **Comment:** The reported superior accuracy and ROC-AUC scores of XGBoost are promising, but it would be useful to include specific numerical values or comparisons to better illustrate the magnitude of the improvement. Additionally, discussing the practical implications of these results in clinical settings could strengthen the argument for their utility.
   - **Response:** We appreciate your constructive feedback. In response to your suggestion, we have updated the manuscript to include specific numerical values for the accuracy and ROC-AUC scores of the XGBoost model, along with comparisons to other models. This enhancement helps to better illustrate the magnitude of the improvement offered by XGBoost. Furthermore, we have expanded the discussion section to explore the practical implications of these results in clinical settings, highlighting how the superior performance of XGBoost could enhance clinical decision-making and improve patient outcomes. This addition aims to strengthen the argument for the utility of our approach in real-world healthcare scenarios.

4. **Comparison of Predictive Biomarkers:**

   - **Comment:** The identification of key predictive biomarkers is valuable, but the authors could benefit from a brief discussion on how these biomarkers compare with existing clinical indicators for CKD. This would provide context on how these findings might impact current diagnostic practices.
   - **Response:** Thank you for your insightful suggestion. We have expanded the discussion section to include a comparison between the key predictive biomarkers identified in our study and the established clinical indicators for CKD, such as serum creatinine and estimated glomerular filtration rate (eGFR). This additional discussion provides a clearer context on how our findings align with or enhance current diagnostic practices. Specifically, we have highlighted how the integration of these biomarkers with traditional indicators could potentially improve early detection and risk stratification in CKD, thereby offering a more comprehensive approach to patient management. These updates have been incorporated in the revised manuscript.

5. **Benchmarking and Impact:**

- **Comment:** The claim that this research sets a benchmark for machine learning tools in personalized healthcare is ambitious. It would be beneficial to provide specific examples or comparisons with existing benchmarks to substantiate this claim and demonstrate the study's impact on the field.
- **Response:** Thank you for your valuable feedback. We have revised the manuscript to include specific examples and comparisons with existing benchmarks in CKD prediction. These additions in the "Related Work" and "Results" sections provide numerical comparisons and demonstrate how our research aligns with and, in some cases, surpasses current benchmarks. This supports our claim of setting a new standard in personalized healthcare.

We would like to thank the reviewers and the Academic Editor once again for their valuable feedback and thoughtful suggestions. We believe the revisions made have significantly improved our manuscript, and we look forward to your further evaluation.

Sincerely,

El Mehdi Chouit
