# Supplementary material for: Interpretable machine learning for chronic kidney disease prediction: Insights from SHAP and LIME analyses
Source: PLoS One. 2026 Feb 26;21(2):e0343205. doi: 10.1371/journal.pone.0343205 (PMC12944776; doi:10.1371/journal.pone.0343205)
Supplement: Attachment — Submitted filename: Response to Reviewers.pdf [file pone.0343205.s002.pdf]

Manuscript Title: Interpretable Machine Learning for Chronic Kidney Disease Prediction: Insights from SHAP and LIME Analyses
Manuscript ID: PONE-D-24-28794

Dear Editorial Team,

We would like to express our sincere appreciation for the thorough and constructive feedback provided on our manuscript. We have carefully considered each comment and have made corresponding revisions to enhance the quality and clarity of our work. Below, we provide a detailed, point-by-point response to each of the reviewers' comments.

Response to Reviewer #1

Comment 1: Ensure the manuscript maintains consistency in terminology. For example, if "CKD" is used throughout, avoid alternating with terms like "kidney disease" without clear contextual purposes.

Response: Thank you for pointing out the importance of maintaining consistent terminology throughout the manuscript. We have carefully reviewed and revised the manuscript to ensure that "CKD" is consistently used as the primary term, avoiding alternation with "kidney disease" or "chronic kidney disease" unless explicitly required for context or clarity. For example, instances of "kidney disease" in the Discussion section and other parts of the text were replaced with "CKD" where appropriate. These revisions enhance the clarity and cohesiveness of the manuscript.

Comment 2: Add this: How a clinician might use SHAP and LIME insights to adjust patient management strategies (e.g., how a high SHAP value for hemoglobin could prompt further diagnostic tests or a change in medication).

Response: We have integrated specific examples to illustrate how clinicians might use SHAP and LIME insights for patient management. For instance, we explain that a high SHAP value for hemoglobin could prompt additional diagnostic tests to detect underlying anemia or necessitate adjustments in medication regimens. These additions are included in the *Future Directions and Real-World Applications* section (Page 21).

Comment 3: There is nothing mentioned about Interoperability Limitation, how SHAP or LIME impact real-world application.

Response: Thank you for raising this critical point. We have added a detailed discussion of interoperability limitations and how SHAP and LIME impact real-world applications. Specifically, we address:

- **User-Friendly Visualizations:** SHAP and LIME outputs must be translated into intuitive formats (e.g., bar charts or contribution plots) that clinicians can interpret with ease.
- **Integration Challenges:** Seamless integration into electronic health records (EHR) systems demands compatibility with existing platforms and workflows.
- **Training and Trust:** Clinicians need training to interpret these tools, and building trust in their outputs is essential.

These points are discussed in detail in the *Future Directions and Real-World Applications* section (Page 23).

Comment 4: Manuscript could suggest how clinicians might visualize SHAP values within electronic health records (EHRs) to make real-time decisions.

Response: We appreciate this insightful suggestion. In the revised manuscript, we propose the following visualization methods for SHAP values within EHR systems:

- **Color-Coded Bar Graphs:** Key features influencing CKD risk could be displayed as color-coded bars for quick identification.
- **Dynamic Contribution Charts:** Interactive charts could link SHAP values to clinical actions, such as ordering diagnostic tests or modifying medication.
- **Real-Time Alerts:** EHRs could generate alerts based on SHAP or LIME insights, prompting timely interventions.

These enhancements are discussed in the *Future Directions and Real-World Applications* section (Page 23).

Comment 5: IF POSSIBLE THEN ADD OTHERWISE LEAVE IT: Visual examples of how SHAP or LIME interpretations were used to make specific clinical decisions (e.g., predicting CKD onset) could provide a clearer link between the technical approach and clinical relevance.

Response: To address this comment, we have included visual examples of SHAP and LIME analyses:

- **SHAP Analysis:** SHAP summary and dependence plots (Figures 4–5) demonstrate the global importance of features such as hemoglobin and eGFRBaseline in predicting CKD onset.
- **LIME Analysis:** LIME plots (Figures 6a–6b) provide local explanations for individual predictions, showcasing how features like sBPBaseline contribute to specific patient outcomes.

These examples illustrate the model's clinical applicability and align with the reviewer's comment. Please refer to the revised manuscript (Pages 18–20).

Comment 6: The authors could further discuss how real-world variability, such as changes in data collection protocols across different clinics or regions, might affect model reliability and ways to handle such scenarios.

Response: We appreciate this comment highlighting a crucial aspect of model reliability. In response, we have expanded the *Future Directions and Real-World Applications* section to address this issue. Specifically, we discuss:

- **Standardization of Data Sources:** Harmonizing data collection practices across clinics and regions to improve consistency.
- **Advanced Imputation Techniques:** Mitigating the effects of missing or incomplete data through robust imputation.
- **Validation Across Diverse Populations:** Testing models across varied geographic and demographic contexts to enhance generalizability.

These additions underscore the importance of addressing real-world variability to ensure robust and reliable predictions (Page 23).

Response to Reviewer #2

Comment: The revision done was not highlighted in the manuscript. It is not the right approach to submit a revision. The revision must be supported by proper highlighting along with page number, section number, where changes were done with respect to the reviewers' comments.

Response: We appreciate the reviewer's feedback regarding the clarity and traceability of revisions. To address this concern, we have taken the following steps:

- **Highlighting Revisions in the Manuscript:** All changes made in response to reviewer comments have been highlighted in the revised manuscript using color-coded markers for easy identification.
- **Annotated Manuscript:** An annotated version of the manuscript, with inline comments linked to reviewer feedback, has been submitted alongside the revised document.

Examples of highlighted revisions include:

- **Comment 3:** Addressed in the *Future Directions and Real-World Applications* section (Page 23), discussing standardization and multimodal approaches.
- **Comment 4:** Enhanced descriptions of SHAP and LIME visualizations in the same section (Page 23).
- **Comment 6:** Expanded discussions on the impact of regional variability in the *Limitations and Future Directions* section (Page 23).

We believe these measures ensure transparency and facilitate a thorough evaluation of the revised manuscript.

Response to Reviewer #5

Comment 1: The abstract needs more improvement. The authors should mention the results and improvements over existing methods.

Response: Thank you for highlighting this important point. We have revised the abstract to include key performance metrics of our proposed model and highlight its improvements over existing methods. Specifically, we now detail the predictive accuracy (e.g., XGBoost achieving 99.5% accuracy and near-perfect ROC-AUC scores for Dataset 1) and improvements in interpretability facilitated by SHAP and LIME. These additions make the abstract more comprehensive and impactful. Please refer to the revised abstract on Page 1.

Comment 2: The problem statement and motivation of the paper need to be clearly mentioned to provide a clear background to the readers.

Response: Thank you for your valuable suggestion. We have revised the *Introduction* section to explicitly state the problem and motivation behind the research. For example, we emphasize the limitations of traditional CKD prediction models and the need for interpretable machine learning techniques like SHAP and LIME to address the complexities of CKD progression. These revisions provide a clear context and justify the significance of our study. Please see the updated *Introduction* on Page (1-2).

Comment 3: If possible, the authors should add the comparison of the proposed model with existing state-of-the-art models to validate its effectiveness.

Response: We appreciate the suggestion to benchmark our model against existing state-of-the-art methods. In response, we have added a subsection titled *Comparison with Existing State-of-the-Art Models* (Page 22). This section compares our XGBoost-based model with established models for CKD prediction reported in the literature. Metrics such as accuracy, AUC, precision, recall, and F1-score are compared, highlighting our model's superior performance on Dataset 1 and competitive performance on Dataset 2. We believe this addition strengthens the validation of our proposed approach.

Comment 4: I appreciate the valuable use of machine learning in the model. For the readers' concerns, I suggest adding/citing the recent computational models related to SHAP and LIME-based interpretation and machine learning-based models such as AIPs-DeepEnC-GA, StackedEnC-AOP, DeepAVPTP-Pred, iAFPs-Mv-BiTCN, AIPs-SnTCN, and Deepstacked-AVPs in order to provide useful information to the readers.

Response: Thank you for this insightful suggestion. We have reviewed the literature on the recommended models and incorporated their citations and descriptions in the *Related Work* section (Page 3-4). Specifically, we discuss how these advanced computational frameworks, such as AIPs-DeepEnC-GA and StackedEnC-AOP, leverage innovative techniques like genetic algorithms and temporal convolutional networks for robust prediction and interpretation. By situating our work within this broader context, we provide readers with a comprehensive understanding of recent advancements and how SHAP and LIME align with these developments.

Comment 5: How the authors handle the overfitting and generalization of the proposed model.

Response: Thank you for emphasizing this crucial aspect. We address overfitting and generalization in multiple sections of the manuscript:

1. **Cross-Validation for Generalization:** We applied stratified 10-fold cross-validation, ensuring representative class distributions and robust performance estimates.
2. **Regularization Techniques:** XGBoost's built-in regularization parameters (L1 and L2) were fine-tuned through grid search to balance complexity and performance.
3. **Early Stopping:** Training was halted based on validation performance to prevent overfitting.
4. **Explainability to Ensure Meaningful Generalization:** SHAP and LIME analyses validated feature importance, ensuring the model generalized to clinically relevant patterns.
5. **Validation Across Diverse Datasets:** Our model was tested on two datasets with distinct characteristics, demonstrating its adaptability.

These methodologies are detailed in the *Materials and Methods* and *Results* sections . We hope these explanations address your comment comprehensively.

Comment 6: The authors should clearly mention the future directions and real-life applications of the proposed study.

Response: We have added a dedicated subsection titled *Future Directions and Real-Life Applications of the Proposed Study* (Page 23). This section highlights:

1. Future research avenues, such as incorporating multimodal data, conducting longitudinal studies, and validating models across diverse populations.
2. Practical applications, including integrating SHAP and LIME visualizations into EHRs, supporting early CKD detection, and enabling personalized patient care through digital tools.

These additions provide a clear roadmap for future research and real-world implementation, addressing the reviewer's comment effectively.

Comment 7: For the optimal parameters selection of the training model, which strategy was applied?

Response: Thank you for your inquiry. Optimal parameter selection was achieved using a grid search strategy combined with stratified 10-fold cross-validation. This approach systematically evaluated parameter combinations and identified those minimizing validation error while optimizing accuracy and AUC. As this methodology is already detailed in the *Materials and Methods* section, no additional modifications were required. Please refer to Page 12 for details.

Comment 8: What are the limitations of the proposed model?

Response: Thank you for raising this important point. We have added in section titled *Future Directions and Real-Life Applications of the Proposed Study* (Page 23), discussing the following:

- Dependence on dataset quality and variability in collection protocols.
- Limited validation across diverse populations.
- Potential confounding factors and interpretability challenges.

We also propose actionable solutions, such as improving dataset diversity, refining feature selection, and adopting advanced explainability techniques. This addition ensures a balanced discussion of the model's scope and limitations.
