# Supplementary material for: Interpretable machine learning for chronic kidney disease prediction: Insights from SHAP and LIME analyses
Source: PLoS One. 2026 Feb 26;21(2):e0343205. doi: 10.1371/journal.pone.0343205 (PMC12944776; doi:10.1371/journal.pone.0343205)
Supplement: Attachment — Submitted filename: reviewer -3.pdf [file pone.0343205.s003.pdf]

Manuscript Title: Interpretable Machine Learning for Chronic Kidney Disease Prediction: Insights from SHAP and LIME Analyses
Manuscript ID: PONE-D-24-28794

Dear Editor and Reviewers,

We sincerely appreciate the reviewers' thorough and insightful feedback, which has helped us enhance the clarity, rigor, and applicability of our manuscript. Based on these suggestions, we have made substantial revisions to improve the discussion on model integration within Electronic Health Record (EHR) systems, the visualization of SHAP and LIME outputs, and the robustness of data quality across institutions. Below, we provide a point-by-point response detailing our revisions. All changes are highlighted in the revised manuscript for easy reference.

Response to Reviewer #1

Comment 1: Interoperability and Integration with EHR Systems

"Mentioned in the response to Reviewer #3, about integration challenges, but lacks detailed practical implementation strategies."

Response: We acknowledge the importance of providing concrete implementation strategies for integrating our model into Electronic Health Record (EHR) systems. We have expanded the discussion in the *Future Directions and Real-World Applications* section by including:

- **Adoption of Healthcare Data Standards:** Our revised manuscript now discusses how interoperability standards such as HL7, FHIR, and SNOMED CT can facilitate structured data exchange between machine learning models and clinical databases.
- **API-Based Model Deployment:** We propose a practical workflow using RESTful and GraphQL APIs to enable real-time model inference, allowing SHAP and LIME outputs to be seamlessly integrated into EHR systems.
- **User-Friendly Visualizations:** We describe how force plots, bar charts, and contribution plots can be embedded into EHR dashboards to enhance interpretability for clinicians.
- **Security and Compliance Considerations:** The revised discussion now includes details on encryption, access control, and regulatory compliance (e.g., HIPAA, GDPR) for secure deployment.

These additions provide a structured and actionable roadmap for deploying interpretable machine learning models in clinical practice.

Comment 2: Visualization of SHAP and LIME Outputs

"Include more intuitive and detailed visualizations of SHAP and LIME outputs. Visual aids will help clinicians better understand how these models make predictions."

Response: To improve the interpretability of SHAP and LIME outputs, we have:

- **Enhanced Figure Explanations:** Each figure (Figures 4–6) now includes detailed annotations explaining how to interpret SHAP summary plots, dependence plots, and LIME explanations in clinical decision-making.
- **Incorporated Force Plots:** To further illustrate individual-level predictions, we added force plots that highlight the positive and negative contributions of key features in specific cases.

- **Clinical Interpretation Context:** We explicitly link SHAP and LIME insights to medical decision points (e.g., hemoglobin thresholds, blood pressure ranges), illustrating how these explanations can guide personalized treatment strategies.

These refinements ensure that our visualizations are accessible and clinically meaningful for non-expert users.

Comment 3: Discussion on Data Quality and Collection Consistency

"Mentioned briefly in the response to Reviewer #6 but requires deeper analysis and solutions."

Response: We have significantly expanded the discussion on data quality and collection consistency in the *Future Directions and Real-World Applications* section, including:

- **Standardization of Data Collection:** The revised manuscript emphasizes the importance of harmonized data collection protocols across institutions, using international standards (e.g., HL7, FHIR, SNOMED CT) to ensure consistency.
- **Robust Data Imputation Techniques:** We highlight the role of advanced imputation methods such as Multiple Imputation by Chained Equations (MICE) in handling missing data while preserving dataset variability.
- **Cross-Center Validation Strategies:** Our study now underscores the importance of validating models across multiple institutions and diverse demographic groups to enhance generalizability and mitigate potential biases.
- **Continuous Model Monitoring:** We propose periodic retraining and recalibration of the model to account for evolving clinical data and ensure sustained performance.

These measures enhance model robustness and ensure reliable predictions across diverse real-world settings.

Conclusion

We sincerely appreciate the reviewers' insightful comments, which have significantly strengthened our manuscript. The expanded discussions on EHR integration, improved visualization strategies, and enhanced data quality considerations align with the reviewers' suggestions and reinforce the real-world impact of our work. We hope that these revisions satisfactorily address all concerns, and we look forward to your assessment of our resubmission.

Sincerely,
El mehdi Chouit
