# Supplementary material for: Interpretable machine learning for chronic kidney disease prediction: Insights from SHAP and LIME analyses
Source: PLoS One. 2026 Feb 26;21(2):e0343205. doi: 10.1371/journal.pone.0343205 (PMC12944776; doi:10.1371/journal.pone.0343205)
Supplement: Attachment — Submitted filename: Responses to reviewers.pdf [file pone.0343205.s006.pdf]

November 12, 2025

Dr. Julfikar Haider
Academic Editor, PLOS ONE

Subject: PONE-D-24-28794R4 — Response to Reviewers

Dear Dr. Julfikar Haider (Academic Editor) and Reviewers,

We sincerely thank you for the continued evaluation of our manuscript, *"Interpretable Machine Learning for Chronic Kidney Disease Prediction: Insights from SHAP and LIME Analyses"* (PONE-D-24-28794R4), and for the **Minor Revision** decision. We are grateful to Reviewer #5 for confirming that earlier concerns have been addressed and for recommending acceptance.

Below, we respond point-by-point to Reviewer #1 (Dr. Herat Joshi), and we indicate where corresponding changes were made in the revised manuscript.

Reviewer #1 — "Consider harmonized feature subsets for partial external validation. . . "

Response. We appreciate this thoughtful methodological suggestion. As discussed in the manuscript, the two cohorts in our study capture **different clinical signals** by design: the Tawam hospital cohort emphasizes cardio-renal/metabolic risk markers (e.g., eGFR, HbA1c, cholesterol), whereas the UCI CKD dataset emphasizes direct renal indicators (e.g., specific gravity, hemoglobin, serum creatinine). The small core of variables truly shared across both cohorts (e.g., age, blood-pressure/hypertension, diabetes flags) is therefore limited.

We carefully examined a cross-cohort transfer restricted to this minimal shared core, but we judged that reporting such results could be **misleading**: the model would be under-specified relative to the clinically informative features identified by SHAP/LIME in each cohort, and the numbers would not reflect the intended deployment setting. To avoid over-interpretation, we have not included a full cross-cohort analysis on the minimal subset at this time. Instead, we have (i) explicitly acknowledged this point and its implications for transportability and (ii) stated that **prospective, multi-site external validation** with richer overlapping laboratory panels is a key next step. These clarifications are now consolidated in a dedicated **Limitations and Future Research Directions** subsection (Section [6.7]).

Reviewer #1 — ". . . and add a calibration analysis."

Response. We agree that probability calibration is essential for clinical use. We therefore (i) added a brief **Calibration methodology** subsection in Methods (Section 4.6) detailing the OOF/outer-fold protocol, 10-bin reliability diagrams, *Brier score* and *Expected Calibration Error (ECE)*, and (ii) reported the results in a dedicated **Model Calibration Analysis** subsection (Section 5.3) with a summary table and two figures (Table 7; Figures 10–11).

Our key findings from this new analysis are:

- **Dataset 1 (Tawam):** We confirm the expected sensitivity–calibration trade-off.
  - *Without SMOTE*, models were well calibrated (e.g., XGBoost Brier/ECE 0.080/0.030; Table 7).

  - *With SMOTE*, calibration degraded as sensitivity improved (e.g., XGBoost Brier/ECE increased to 0.112/0.129).
  - As shown in Figure 10, this indicates that **site-specific post-hoc recalibration** (e.g., isotonic/Platt) is advisable when oversampling is used in this setting.
- **Dataset 2 (UCI):** Calibration remained **strong** and was minimally affected by SMOTE, consistent with the dataset's high separability.
  - XGBoost Brier scores were excellent in both conditions (0.013 without SMOTE vs. 0.010 with SMOTE).
  - Other tree models (RF, LGBM) also showed strong and stable calibration (Table 7; Figure 11).

All values are derived from *outer-fold OOF predictions* to prevent information leakage. These additions strengthen the manuscript by clarifying the context-dependent impact of SMOTE, and they *do not alter our main conclusions.*

Reviewer #1 — "Add an explicit 'Limitations' subsection (dataset size, single-center data, absence of newer XAI methods)."

Response. Done. We reorganized and consolidated the discussion into a single **Limitations and Future Research Directions** subsection (Section [6.7]), which now explicitly covers: (1) modest sample size and the limited number of progression events in the hospital cohort, (2) single-center nature of the Tawam data and generalizability considerations, (3) the calibration trade-off induced by class balancing (SMOTE) and the potential need for post-hoc recalibration, and (4) the scope of explainability (SHAP for global, LIME for local) while acknowledging that newer XAI paradigms (e.g., counterfactual, concept-based explanations) are planned for future work.

We believe these revisions increase methodological transparency and clinical credibility while preserving the main conclusions of the manuscript. We respectfully submit the revised files: (i) clean manuscript, (ii) tracked-changes version, and (iii) this response letter.

Thank you very much for your consideration.

Sincerely,

El Mehdi Chouit
on behalf of all co-authors
