# Supplementary material for: Interpretable machine learning for chronic kidney disease prediction: Insights from SHAP and LIME analyses
Source: PLoS One. 2026 Feb 26;21(2):e0343205. doi: 10.1371/journal.pone.0343205 (PMC12944776; doi:10.1371/journal.pone.0343205)
Supplement: Attachment — Submitted filename: PONE-D-24-28794_R3_revision.docx [file pone.0343205.s004.docx]

The results for first dataset have major flaws in AUC calculation The models are overfitted and should be diagnosed and resolved. See the further comments

- Figure 3a is highly doubtful! AUC values close to 1.00 with near-zero variance across cross-validation folds is highly suspicious. It may have serious overfitting or data leakage. It suggests that models may have seen test data during training, or some features are strongly correlated with the target.
- Also, the figure 3a has lack of error bars / AUC ≈ 0.00 SD. It suggests that CV folds weren’t truly independent, or that the variance was not computed correctly. Adding F1 score would be beneficial as well once the reason for this overfitting is identified.
- The authors should provide results with and without SMOTE for both datasets
- The model with the first dataset should be validated with the second dataset. This would be an external validation
- Provide a supplementary table by keeping only the selected variables for both datasets
- The scientific contribution of this study is not clear
- What was the rationale of using two datasets with different variables? Can author test these different ML models for two datasets with same variables
- The newly added verbiages should be added as paragraph, not as a bullet point
